# Taste Processing: Insights from Animal Models

**DOI:** 10.3390/molecules25143112

**Published:** 2020-07-08

**Authors:** Andrés Molero-Chamizo, Guadalupe Nathzidy Rivera-Urbina

**Affiliations:** 1 Department of Psychology, Psychobiology Area, University of Huelva, Campus El Carmen, 21071 Huelva, Spain; 2 Faculty of administrative and social sciences, Autonomous University of Baja California, Tijuana 22890, Mexico; nathzidy@hotmail.com

**Keywords:** flavour, molecular signalling, olfactory processing, receptors, taste learning, taste processing

## Abstract

Taste processing is an adaptive mechanism involving complex physiological, motivational and cognitive processes. Animal models have provided relevant data about the neuroanatomical and neurobiological components of taste processing. From these models, two important domains of taste responses are described in this review. The first part focuses on the neuroanatomical and neurophysiological bases of olfactory and taste processing. The second part describes the biological and behavioral characteristics of taste learning, with an emphasis on conditioned taste aversion as a key process for the survival and health of many species, including humans.

## 1. Introduction

Three important sensory systems depend on physical stimuli to initiate their processing. The somatosensory system recognises touch, pain and temperature; the auditory system recognises sound waves and the visual system recognises visual waves, as specific physical modalities. There are two other sensory systems which, however, depend on the stimulation produced by different chemical molecules. Olfactory and taste systems are activated by small particles stimulating specific receptor cells located in the respective membranes of the nose and tongue. Both mechanisms are critical for feeding and are therefore essential for the survival and evolution of species. Food releases odorant volatile particles that stimulate the olfactory system, allowing the recognition of food and the identification of any potential danger. Additionally, different chemical elements of food initiate the activation of the taste system in the lingual epithelium. This mechanism allows the identification of the five basic taste qualities associated with food, that is, sweet, salty, sour, bitter and umami [1,2,3]. Flavour processing involves the interaction of both processes, i.e., taste and odour recognition. Therefore, taste is considered in this review as a sensation caused by chemical compounds released from foods and is originated from the taste buds, then transferred by taste nerves, and finally processed by different brainstem and brain structures.

The neuroanatomy and neurophysiology of the olfactory, flavour and taste systems have been investigated in the laboratory using different animal models [4]. Due to the relevance of these systems for the survival of animals, specific descriptions of the neuroanatomical and neurophysiological fundamentals of olfactory and taste processing are provided in this review. Additionally, considering that taste learning is a critical process for survival [5], we also include a section on associative taste learning, particularly conditioned taste aversion (CTA). Taste learning recruits multiple brain and brainstem mechanisms and molecular pathways, including genetic, receptor, and intracellular and extracellular signalling biological levels. Specific molecular pathways and processes for the acquisition of taste learning and the formation of taste memories have been well identified [6]. However, further molecular, physiological and behavioural studies are needed to elucidate the complete nature of the taste system and the neural and molecular mechanisms of CTA. The knowledge of the molecular bases involved in the acquisition, retention and extinction of CTA may help to understand the brain mechanisms of normal and altered taste learning. Moreover, an understanding of the neurobiology of taste learning, and particularly the neurobiological mechanisms of CTA, could provide valuable information to treat altered eating behaviours in humans, such as anorexia or bulimia, for therapeutic purposes.

## 2. Taste System

**Figure 1:**
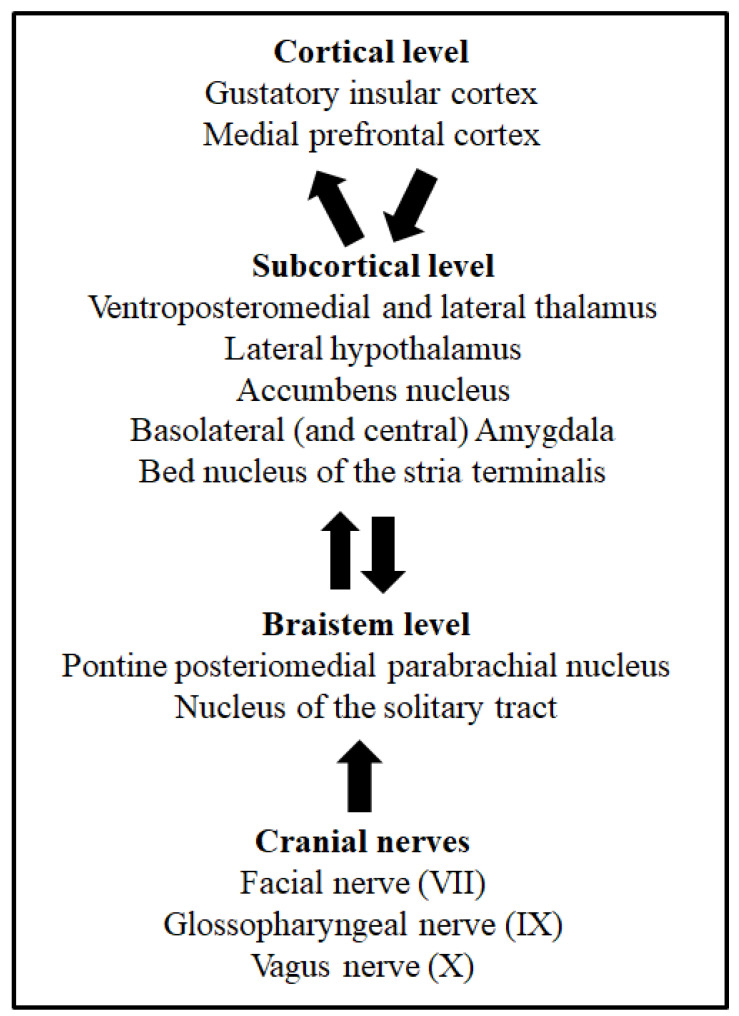
Cortical, subcortical, and brainstem levels of taste processing and taste learning. From bottom to top, arrows indicate the ascending taste pathway from the oral cavity and the afferent and efferent projections associated to taste learning. The cranial nerves involved in taste processing from the oral membranes are also shown.

Taste buds of mammals’ oral cavity membranes contain dozens of clustered taste receptor cells selectively sensitive to one of the five basic taste qualities: sweet, salty, sour, bitter and umami [7]. Taste and visceral processing signals are sent by the VII, IX and X cranial nerves to different nuclei of the brainstem [8]. The nucleus of the solitary tract (NTS) is the first brainstem nucleus that receives taste afferents [8,9,10]. From this nucleus, taste information is ipsilaterally sent to another brainstem group of cells, the posteromedial parabrachial nucleus, and then ascending projections reach several brain structures, such as the lateral hypothalamus, the bed nucleus of the stria terminalis, the amygdala and the ventroposteromedial and lateral thalamus [11,12]. Finally, the higher level of taste processing takes place in the gustatory insular cortex [10,13,14]. This is the taste pathway described in rodents. However, in humans, the taste discrimination process might include mechanisms and systems parallel to taste detection [15]. Figure 1 depicts the main structures and pathways of taste processing in rodents. These systems, together with the pathways involved in visceral malaise processing via the vagus nerve (X cranial nerve), are necessary for taste learning, as well as taste aversion learning and memory [6]. Indeed, the involvement of different taste processing levels through the central nervous system makes possible the modulation of somatic and autonomic responses, as well as the control of the behavioural and hedonic responses of animals [16]. The relevance of taste learning for eating behaviours lies not only in the decision to search for some foods and avoid other foods due to the acquisition of learned taste preferences, but mainly because this decision can be vital, especially in omnivorous species, when food is potentially harmful, as is detailed later. For this reason, taste learning requires special attention.

Additionally, the olfactory system, which is responsible for odour processing, plays an essential role in taste processing and taste learning. Down-top odour processing involves olfactory sensory neurons [17], the olfactory nerve (I cranial nerve) [18], the olfactory bulbs and the olfactory tract [19]. The brain structures that process olfactory information are the piriform cortex, the olfactory tubercle, the periamygdaloid and lateral entorhinal cortices, the cortical region of the amygdaloid nuclei, the ventral tenia teat, the nucleus of the lateral olfactory tract, the anterior cingulate cortex, the insular cortex and the overlying operculum (including the somatomotor mouth region), and the orbitofrontal cortex [18,20]. The relevance of the olfactory system for taste processing and taste learning has been widely described in rodents [21,22].

## 3. Taste Learning

The ability to distinguish between different tastes is a learning related to survival. For example, the sweet taste of saccharin or glucose, which provides high calories and therefore energy [23,24], induces dopamine release in the reward system of the brain [25,26]. This system makes it possible to learn taste preferences as an adaptive mechanism in nature. Animal models have revealed numerous molecular processes and brain areas involved in taste learning [27]. Due to the critical adaptive function of the acquisition of taste aversion, this section focuses on the neuroanatomy and physiology of this type of taste learning.

### 3.1. Conditioned Taste Aversion: A Peculiar Taste Learning

Vertebrates and some invertebrates have developed neural systems that allow the association of non-familiar tastes with visceral toxic effects, resulting in a learned aversion (known as conditioned taste aversion or CTA) to tastes with similar taste characteristics [10]. The learned aversion results in an adaptative response of rejection to the toxic food that induced gastrointestinal malaise (thus reducing the probability of toxic experiences) and an acquired perception of unpleasant taste. This type of taste learning is useful for preserving life, particularly for omnivorous species, including human beings. The acquired taste aversion can be extinguished if further experiences with the same taste stimulus (or even similar taste stimuli) are not associated with poisoning or negative consequences.

CTA and CTA extinction can be reproduced in the laboratory with animal models in order to explore the neurobiological and behavioural characteristics of this learning [28,29]. In the laboratory (as in nature), CTA is characterised by the fact that taste aversion can be acquired through a single association between the taste stimulus and visceral malaise, and because the association between the novel taste and visceral consequences is possible even some hours after the processing of both stimuli [30,31]. This characteristic of CTA, to resist long delays between stimuli, is explained by the physiological processes of digestion, which implies a delay between the taste processing and visceral consequences. Considering this physiological time interval between the taste processing and the gastrointestinal malaise, CTA could result from the association between the taste memory’s trace and the visceral disease. The CTA paradigm used in the laboratory is a useful tool to investigate the biological and behavioural bases of aversive learning and taste memory, not only regarding the specificity of CTA but also with respect to other taste learning phenomena, such as neophobia (a precautionary response to the first exposure to a novel food or taste) or latent inhibition of CTA (a reduced conditioned aversion resulting from taste pre-exposures without negative or aversive consequences) [32,33,34,35]. An example of the relevance of the CTA paradigm is the finding that physical (external) and internal (such as the time of day) cues can separately modulate the magnitude of a learned aversion [36,37,38].

**Figure 2:**
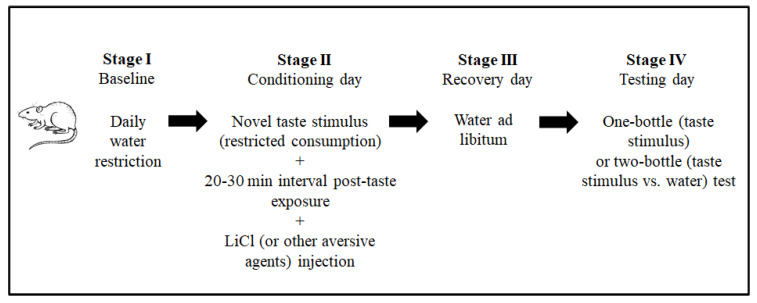
Principal stages of the procedure to induce conditioned taste aversion (CTA) in laboratory animal models.

The conventional CTA paradigm includes ad libitum feeding throughout the procedure and a daily water restriction baseline stage to facilitate similar physiological states among the animals. The next stage is the conditioning session. In this stage, the animals are exposed to a novel taste stimulus (the conditioning stimulus, CS, in the terminology of associative learning). In the CTA paradigm, taste stimuli are usually provided as fluids (for example, as saccharin or sodium chloride solutions) to facilitate intake measurement. Around 20 min after intake, an intraperitoneal injection of lithium chloride (LiCl 0.15 M, 2% of body weight) is administrated to induce gastrointestinal malaise (the unconditioned stimulus, US). To a lesser extent, other aversive stimuli have also been used to induce taste aversion [39,40]. A recovery day with ad libitum water usually follows this stage. The magnitude of the taste aversion is evaluated the next day (testing day), by one-bottle (CS) or two-bottle (CS vs. water) tests, according to the amounts consumed. Figure 2 shows the CTA paradigm stages. This paradigm is used to explore the neurobiology of taste aversion learning and memory.

### 3.2. Neural Network of CTA

A complex nervous system mechanism is involved in the acquisition of CTA [41,42]. Animal models provide critical data to understand the neuroanatomy and neurobiology of taste learning and memory. The neural network of taste aversion learning and memory includes the nucleus of the solitary tract (NTS), the posteromedial pontine parabrachial nucleus, the lateral hypothalamus, the bed nucleus of the stria terminalis, the amygdala and the ventroposteromedial and lateral thalamus. The superior cortical level of processing has been described in the gustatory insular cortex region. The functional connectivity between the parabrachial nucleus and the gustatory insular cortex is selectively involved in the acquisition of CTA but not in the formation of safe taste memories [43]. These pathways and the vagal system involved in the processing of visceral malaise are necessary for the acquisition of CTA and taste aversion memory [10,11,12,14]. Moreover, the role of other brain structures in the neurobiology of CTA, such as the medial prefrontal cortex and the nucleus accumbens, is being elucidated at present [44], together with the functions of the piriform [45] and perirhinal [46] cortices in taste recognition. The neural system of CTA involves the activity of this brain and brainstem network, but the specific functions of each component are not fully understood. Although the CTA mechanisms of the gustatory insular cortex and parabrachial nucleus are well described, the involvement of the amygdala and its nuclei in specific processes of taste aversion learning and memory is not fully known [47,48,49]. Animal lesion studies have pointed to the central and basolateral nuclei of the amygdala as the amygdaloid nuclei with specific functions in taste aversion learning and memory [50]. However, the basolateral amygdala seems to be the main nucleus involved in the acquisition of CTA [49], probably modulating the magnitude of taste aversion [51,52]. A possible mechanism by which basolateral amygdala can modulate the intensity of CTA is through the neophobia phenomenon, considering that this nucleus is implicated in the perception of novelty of taste stimuli. The correct processing of novelty is one of the mechanisms affecting the magnitude of CTA [53]. In addition to lesion studies, other methods and approaches have also pointed to the basolateral amygdala as a selective amygdaloid nucleus mediating the acquisition of CTA. By two-photon calcium imaging it has been revealed that a CTA-dependent neuronal activation of specific neurons of the insular cortex that project to the basolateral amygdala [54], and chemical activation of the insular cortex-basolateral amygdala projection by Clozapine-*N*-oxide after taste exposure, can induce aversive taste memory in mice [55]. Thus, the function of the basolateral amygdala on CTA might be controlled by afferent axons from the gustatory insular cortex [53]. Moreover, molecular studies have supported the relevance of this cortico-amygdaloid projection for the formation of CTA [56,57]. It can be concluded that specific connections between the gustatory insular cortex and the basolateral complex of the amygdala [49,57,58], and between the amygdala and the brainstem nuclei involved in CTA [12,47,48], could be recruited to influence the intensity of acquired taste aversions.

### 3.3. Molecular Mechanisms of CTA

Some molecular mechanisms are specific to certain forms of taste learning and memory [59]. However, learning of novel tastes, taste familiarity and taste aversion extinction share biological pathways and mechanisms with CTA. The transcriptional processes necessary for the acquisition of taste learning or processing of taste novelty that occur in the gustatory insular cortex have been described in rats [60]. Particularly, this model has provided relevant information about how novel taste experience, a process that strengthens the acquisition of CTA, modifies the genetic transcription in this cortical area during taste memory consolidation [60]. Likewise, taste learning of novel or familiar tastes promotes different changes in the transcriptome of this cortical region. Moreover, the consolidation of positive or negative taste learning (according to its positive or negative visceral consequences) also induces transcriptional activity in this region [60]. Learning of novel tastes induces biochemical alterations in the gustatory insular cortex of other rodents as well, including increased cholinergic activity [61], and changes in protein phosphorylation [62], facilitating taste memory consolidation [63,64]. Furthermore, several studies have shown that taste memory consolidation is altered after pharmacological inhibition of protein synthesis in the gustatory insular cortex [65,66]. Recently [67], the administration of protein synthesis inhibitors directly into the gustatory insular cortex during long-term taste memory acquisition altered the formation of long-term memory. However, this procedure did not affect memory persistence when these inhibitors were infused 3 days after the memory formation. Interestingly, the infusion of protein synthesis inhibitors 14 days after the memory acquisition increased the memory persistence [67], which suggests that long-term memory may be altered by protein synthesis inhibitors, even several days after the formation of taste memory.

Other molecular mechanisms of taste learning that could participate in CTA have been seen in the gustatory insular cortex. Various immediate early genes identified in this cortical region during taste learning, such as the activity-regulated cytoskeleton associated protein (Arc)/Arg3.1 gene, seem to regulate the excitability of synapses associated with synaptic plasticity processes and long-term taste memory [68,69]. The function of the Arc/Arg3.1 protein appears to be different depending on the specificity of taste learning, since novel taste learning can increase as well as reduce the expression of this protein in the gustatory insular cortex, according to specific time points. These transcriptional changes may last for hours and are more intense compared to the processing of familiar tastes [60]. Moreover, a hemispheric lateralisation of the expression of Arc/Arg3.1 protein is observed in the gustatory insular cortex related to the processing of novel tastes [70].

The neural plasticity mechanisms related to taste learning, including CTA, also involve other molecular sequences. The expression of brain-derived neurotrophic factor (BDNF) in the gustatory insular cortex (and the basolateral amygdala) induces long-term synaptic plasticity, and the acquisition of taste aversions seems to block the long-lasting BDNF-induced strengthening of synaptic plasticity [71]. These findings point to the BDNF gene expression in the gustatory insular cortex as one of the molecular mechanisms critically involved in the long-term synaptic plasticity processes related to taste memory. Additional gene expressions have been found in the gustatory insular cortex during taste learning, including the expression of c-fos [72], Homer1a [73] and the transcription factor Elk-1 [74]. The functions of these gene expressions and their respective proteins are unclear, although it is assumed that they are a relevant part of the synaptic plasticity necessary for taste learning and memory [75]. All this evidence indicates that the gene expression in the gustatory insular cortex and in other brain regions involved in taste learning is a molecular key for the acquisition of taste learning and the subsequent taste memory [63].

The neurobiology of taste learning includes molecular pathways particularly associated with the acquisition of CTA. Animal models and numerous in vitro methods are significantly contributing to the discovery of the molecular mechanisms of taste aversion learning and memory. The intracellular and extracellular signalling mechanisms observed in CTA reveal plasticity processes involving gene expression. Thus, taste learning and taste memory consolidation have been related to specific protein expression in the gustatory insular cortex [65,66,67]. Gene expression of BDNF in the gustatory insular cortex and basolateral amygdala has been described during the long-term synaptic plasticity associated with taste aversion memory [71]. Receptor expression is another plasticity process necessary for the acquisition of CTA. The *N*-methyl-D-aspartate glutamate (NMDA), α-amino-3-hydroxy-5-methyl-4-isoxazolepropionic acid glutamate (AMPA), and GABA-A receptors, as well as the muscarinic receptor of acetylcholine (mACh), are some of the neurotransmitter receptors involved in the formation of CTA [56,75,76]. Calcium calmodulin-dependent protein kinase IIα (CaMKIIα), protein kinase A (PKA) and C (PKC), cyclic adenosine monophosphate (cAMP) and adenylyl cyclase are also specific molecules involved in the plasticity processes initiated by taste aversion learning and memory [6]. Table 1 shows proteins, receptors and other molecules observed in regard to taste learning and CTA.

Regarding neuronal signalling and plasticity associated with taste aversion learning and memory, recent molecular studies have shown several intracellular and extracellular signalling pathways. Increased activity of the *N*-ethylmaleimide-sensitive factor (NSF), an active protein involved in the membrane fusion process through soluble *N*-ethylmaleimide-sensitive factor attachment protein receptors (SNAREs), has been found in the perirhinal cortex and the basolateral amygdala during habituation of taste neophobia and taste recognition memory. The neuroplasticity and memory processing triggered by NSF in these brain structures are mediated by cellular signalling pathways, including the extracellular activation of GluR2 subunit-containing AMPA receptors [90].

Intracellular signalling linked to taste aversion learning and memory requires multiple molecular processes. Exposure to novel tastes activates dopamine receptors in the gustatory insular cortex. This activation increases cAMP levels through the activation of adenylyl cyclase, in turn activating cAMP-dependent PKA [76]. Then, PKA promotes cAMP response element-binding protein (CREB) phosphorylation and gene expression related to synaptic plasticity and long-term taste memory. Additionally, taste aversion memory consolidation has been associated with the stimulation of mACh receptors and PKC activation and an increased activity of the extracellular signal-regulated kinases (ERK) [76]. With respect to visceral processing, gastrointestinal signals trigger CaMKII and PKA activation and CREB phosphorylation, which also seems to promote protein synthesis [76].

BDNF has been identified as another molecule with a critical function in CTA neural signalling. In rats, intracortical microinfusion of BDNF in the gustatory insular cortex reduces taste aversion intensity and enhances the extinction of CTA [78]. BDNF reduces CTA even if administered 10 days after the acquisition of taste aversion, which suggests that the activity of this molecule in this cortical area is critical for the expression of CTA several hours after association is established between CS and US [85]. When BDNF is administered in the gustatory insular cortex before the CTA acquisition, the retention of taste aversion is enhanced [86], which reveals the existence of different mechanisms of this molecule depending on the learning stage. The functional effects of the BDNF signalling on the gustatory insular cortex have been linked to TrkB receptor activity because specific effects of this molecule were blocked by the administration of K252a, an antagonist of this type of receptor [78].

In relation to CTA memory extinction, elaborate studies have revealed specific signalling molecular mechanisms during structural synaptic plasticity. Actin rearrangement is a structural change mechanism that strengthens synapses. Actin rearrangement and increased synaptic density have been found in the infralimbic cortex during the extinction of CTA memory [87]. More specifically, the structural plasticity of CTA memory extinction involves myosin II phosphorylation. Administration of myosin II ATPase inhibitors into the infralimbic cortex blocks the actin rearrangement mechanism and the subsequent CTA memory extinction [87]. These findings suggest that increased myosin II in the infralimbic cortex causes structural changes that modulate the appearance of new synapses through structural plasticity mechanisms during taste aversion memory extinction. CTA memory extinction also requires other specific signalling processes, such as the activation of the D2 receptor [92] and the intracellular phosphoinositide 3-kinase (PI3K) signalling through the phosphorylation of the isoform-specific AKT kinase, which is increased after the acquisition of CTA and decreased after the extinction. Therefore, PI3K signalling could be a molecular process necessary for the consolidation of aversive taste memory [91].

In regard to learning of novel tastes, there are other plasticity processes involving the NMDA receptor and intracellular protein signalling. As part of these processes, increases in the PSD-95 protein postsynaptic density and phosphorylated NR2B subunit of the NMDA receptor have been described in the gustatory insular cortex [77]. Since both novel taste learning and CTA require novel taste processing, their respective plasticity processes may share molecular mechanisms.

As mentioned above, receptor expression is another synaptic plasticity mechanism of taste learning and CTA, and specific neurotransmitter and receptor systems are being investigated in this context. The acquisition of CTA seems to require the activation of glutamatergic receptors, such as AMPA and NMDA, as well as muscarinic receptors in the gustatory insular cortex. The synaptic plasticity of CTA is apparently related to tyrosine phosphorylation of the NR2B subunit of the NMDA receptor in this cortical region [84,93]. The synaptic plasticity associated with the CTA acquisition also involves the activity of the NR1 subunit of the NMDA receptor in the prefrontal cortex [44]. Considering that the associative processes of CTA occur several hours after the novel taste experience, the NMDA receptor-dependent plasticity during this learning could be evident at different time points. For instance, the proteasome activity in the gustatory insular cortex is increased 4 h after the exposure to a novel taste. This effect is dependent on the NMDA receptor and the CaMKII signalling during CTA, which indicates that CTA acquisition requires NMDA receptor-dependent proteasome activity in this cortical region [94]. Moreover, the activity of NMDA receptors in the basolateral amygdala-gustatory insular cortex projection necessary for CTA memory involves PKC and PKA signalling [88]. Interestingly, the NMDA receptor and PKC, but not PKA, signals are molecular mechanisms observed in the long-term plasticity of the gustatory insular cortex during CTA. In addition, the NMDA receptor and PKA and PKC phosphorylation in the gustatory insular cortex during visceral processing are molecular mechanisms that are concurrent with taste processing during CTA acquisition [76].

The acquisition, but not the retrieval, of novel taste memories involves the activity of metabotropic glutamate receptors and β-adrenergic and dopamine receptors in the gustatory insular cortex [82]. In particular, exposures to novel tastes increase the phosphorylation of the NR2A and NR2B subunits of the NMDA receptor in the gustatory insular cortex [83]. The NMDA receptors participating in novel taste processing depend on dopaminergic signalling pathways. Tyrosine phosphorylation of the NR2B Y1472 subunit of the NMDA receptor depends on the activity of the D1 receptor. This phosphorylation is critical for the ERK 1/2 activation during novel taste stimuli processing [84]. Two parallel gustatory memory traces for novel tastes have been identified in rats. One of them is a short-duration robust trace in the gustatory insular cortex (active for about 3 h) that is regulated by a CaMKIIα-AMPA glutamate receptor pathway (which in turn depends on the NMDA receptor). The other is a long-duration trace (estimated in 8 h) that depends on the short-duration trace [89]. On the other hand, the NMDA receptor-dependent upregulation of proteasome activity in the gustatory insular cortex that is linked to novel taste learning is an essential molecular mechanism for the association of novel tastes with malaise during the CTA acquisition [79]. However, a reduced proteasome-mediated degradation has been found in the gustatory insular cortex 20 min after the exposure to a novel taste, and this effect was mACh-dependent [79]. Therefore, the reduced proteasome-mediated degradation in the gustatory insular cortex after the processing of novel tastes is mediated by mACh, but not NMDA, receptors. Memory processes for taste familiarity could thus involve mACh-dependent reduced proteasome activity [79]. Finally, taste-recognition memory, including taste neophobia (a first step for the acquisition of CTA), is related to GABAergic and cholinergic neurotransmission through GABA-A and mACh receptors [75,80,81].

## 4. Conclusions

Olfactory and taste processing (i.e., flavour processing) is a critical mechanism for taste learning. The acquisition and memory of taste aversion is a type of associative learning that prevents further exposures to toxic foods. This adaptive learning can be reproduced in the laboratory to study the behavioural and biological fundamentals of taste learning and CTA acquisition, memory and extinction. Animal models are providing valuable insights into the characteristics and nature of taste learning by lesion procedures, immunohistochemistry, genetic engineering, molecular methods, brain images, behavioural studies, etc. The findings of these studies are helping to identify the neurobiological and behavioural mechanisms of normal and altered taste learning and memory, including CTA. Particularly, the molecular pathways that allow the acquisition and memory of CTA are being discovered. These include gene expression, a multitude of neurotransmitter receptors, and complex intracellular and extracellular signalling mechanisms. The expression of specific genes promotes the synaptic plasticity necessary for taste learning and CTA acquisition. The molecular mechanisms of taste learning and CTA acquisition and extinction with the most evidence are those involving the mACh, AMPA and NMDA neurotransmitter receptors. The cellular signalling triggered by these receptors and molecular mechanisms promotes structural and functional plasticity changes that facilitate taste learning and CTA. Despite this remarkable knowledge, much remains to be discovered about the detailed functioning of all taste learning and memory systems. Specifically, future studies are expected to map out the complete molecular network of taste learning and memory, as well as CTA.

## Figures and Tables

**Table 1:** Proteins, receptors and other molecules involved in taste learning. Brain regions and their functions related to these molecular processes are shown in parentheses.

Receptors	Protein Expression	Molecular Signals
NMDAr (GIC; CTA acquisition and taste learning) [56,75,76,77] AMPAr (GIC; CTA acquisition) [56,75,76] GluR2 (amygdala and PEC; SP and taste memory) [78] mACh (GIC; neophobia, SP and CTA acquisition) [56,75,76,79,80,81] DA (GIC; novel taste memory) [76,82] GABA-A (GIC; taste recognition) [56,75,76,80,81] NR1 (PFC; SP in CTA acquisition) [44] NR2A-2B (GIC; CTA acquisition and taste processing) [83] TrkB (GIC; taste memory) [78] β-adrenergic (GIC; novel taste memory) [82] D1 (GIC; novel taste processing and memory) [84]	c-fos (GIC; SP and LT taste memory) [72] BDNF (GIC and amygdala; SP in taste memory and CTA) [71,78,85,86] Homer 1a (GIC; SP and LT taste memory) [73] Arc/Arg3.1 (GIC; CTA memory) [60,68,69,70] Transcription factor Elk-1 (GIC; SP and LT taste memory) [74]	cAMP (GIC; SP and taste memory) [76] Adenylyl cyclase (GIC; SP and taste memory) [76] Actin (IFC; structural plasticity and CTA memory) [87] PKA (GIC and amygdala CTA memory) [76,88] CaMKIIα (GIC; SP, novel taste memory, CTA acquisition) [89] PKC (GIC and amygdala; LT plasticity and CTA memory) [76,88] NSF (amygdala and PEC; SP and taste memory) [90] ERK1/2 (GIC; novel taste processing, SP and taste memory) [82,84] Myosin II (IFC; structural plasticity and CTA memory) [87] PSD-95 (GIC; SP and taste learning) [64,77] PI3K (GIC; SP and CTA memory) [91]

PUAMPAr, α-amino-3-hydroxy-5-methyl-4-isoxazolepropionic acid glutamate receptor; (Arc)/Arg3.1, activity-regulated cytoskeleton associated protein/Arg3.1, immediate early gene (IEG); BDNF, brain-derived neurotrophic factor; CaMKIIα, calcium calmodulin-dependent protein kinase IIα; CTA, conditioned taste aversion; DA, dopamine receptor; ERK, extracellular signal-regulated kinases 1/2; GIC, gustatory insular cortex; GluR2, GluR2 subunit-containing AMPA receptor; IFC, infralimbic cortex; LT, long-term; mACh, muscarinic acetylcholine receptor; NMDAr, *N*-methyl-D-aspartate glutamate receptor; NR1-2B-2A, subunits of the NMDA receptor; NSF, *N*-ethylmaleimide-sensitive factor; PEC, perirhinal cortex; PFC, prefrontal cortex; PI3K, phosphoinositide 3-kinase; PKA, protein kinase A; PKC, protein kinase C; PSD-95, NR2B-associated protein; SP, synaptic plasticity; TrkB, BDNF receptor. Modified from [5], with permission from the Editors.
